# First Images on a Next-Generation Extended–Field-of-View Digital Bismuth Germanium Oxide Total-Body PET/CT

**DOI:** 10.2967/jnumed.125.271995

**Published:** 2026-08

**Authors:** Michael S. Hofman, Alicia Corlett, Cameron Pain, Price Jackson, Ramin Alipour, Grace Kong, Aaron J.N. Wong, James Buteau, Tim Akhurst, Dulash Fernando, Shankar Siva, Declan G. Murphy

**Affiliations:** 1 Peter MacCallum Cancer Centre, Melbourne, Victoria, Australia; and; 2 University of Melbourne, Melbourne, Victoria, Australia

A ^18^F-DCFPyL PET/CT was performed for biochemical recurrence (prostate-specific antigen level, 8.0 ng/mL, with a doubling time of 1 y and involved surgical margin) in a patient with Gleason grade group 3 prostate cancer, 12 y after radical prostatectomy. After injection of 341 MBq, imaging was acquired on a standard–field-of-view (SFOV; 26 cm) PET/CT equipped with 20-mm-thick lutetium oxyorthosilicate (LSO) crystals and digital detectors (Biograph VISION 600; Siemens Healthineers). PET data were reconstructed using point-spread function and time-of-flight data (acquisition time, 9 min 20 s; 440 × 440 matrix).

The acquisition was immediately repeated on a 128-cm long–axial-field-of-view (LAFOV) PET/CT scanner featuring 30-mm-thick bismuth germanium oxide (BGO) detectors with a silicon photomultiplier (Omni 128; GE HealthCare) as part of a clinical study (NCT07263815). Images were reconstructed with block-sequential regularized expectation maximization (Q.Clear; GE HealthCare), with a *b* value of 100, high-precision deep learning, and a 384 × 384 matrix.

The uptake time for the first acquisition was 70 min for SFOV and 90 min for LAFOV. Physiologic liver and parotid uptake were comparable (3.9 vs. 4.0 and 16.4 vs. 16.9 for SFOV and LAFOV, respectively). On the LAFOV PET/CT, an initial dynamic acquisition at the time of radiotracer injection and a delayed acquisition at 2 h 37 min was also obtained.

There was intense uptake in a small area of recurrence in the prostatectomy bed near the urethra (SUV_max_, 44 on SFOV vs. 73 on LAFOV) (Fig. 1). A second focus (<5 mm) indicating recurrence adjacent to a surgical clip near the left seminal vesicle bed was visualized only on the LAFOV PET/CT (SUV_max_, 2 [not appreciably abnormal] with SFOV vs. 20 with LAFOV PET/CT). Variable bed-step reconstructions on LAFOV PET/CT–acquired images at 30 s and 1, 2, 3, and 5 min visualized the smaller recurrence with confidence from an acquisition time of 2 min or greater. Both areas intensified on delayed LAFOV PET/CT acquisition (SUV_max_, 94 and 32, respectively). Only the larger focus was seen on the dynamic acquisition before physiologic bladder accumulation.

**FIGURE 1.**
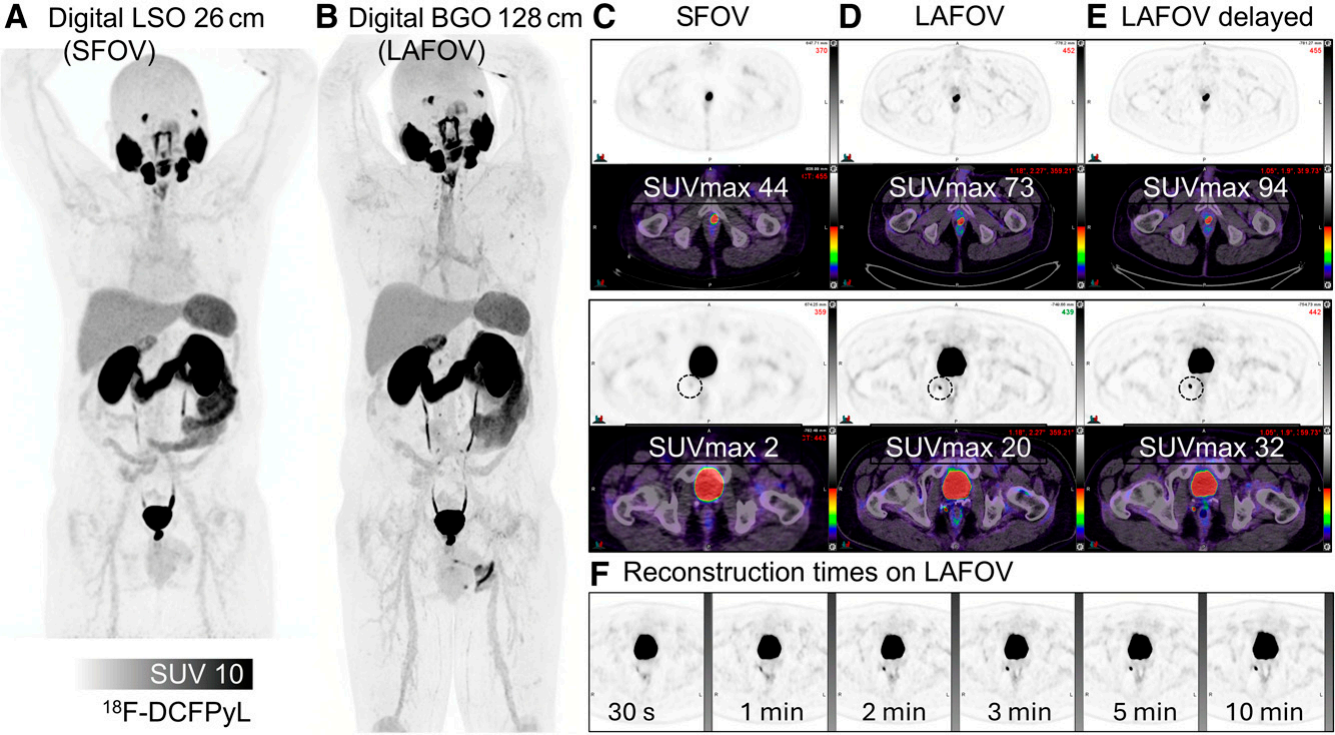
(A and B) Maximum-intensity-projection images on SFOV and LAFOV PET/CT, showing better visualization of stellate and lumbar ganglion and submucosal tracheal physiologic activity on LAFOV PET/CT. (C–E) Top: recurrence with intense uptake on all images. Bottom: <5-mm recurrence visible only on LAFOV. (F) Variable reconstruction times demonstrate clear visualization on acquisitions at 2 min and later.

The supplemental video, available at http://jnm.snmjournals.org, shows rotating maximum-intensity-projection and dynamic images of the local recurrence before physiologic bladder accumulation, demonstrating the superiority of LAFOV PET/CT for identifying pelvic recurrence of prostate cancer.

Informed by total-body PET findings, salvage radiotherapy to the prostate bed was planned, dose-painted with a reduced dose to the remaining prostate fossa and a microboost to areas of macroscopic disease seen on LAFOV PET/CT.

Additionally, multilevel physiologic stellate and lumbosacral ganglia and physiologic submucosal tracheal activity were better visualized on the LAFOV PET/CT.

Previous data demonstrated superiority of the digital BGO to LSO systems, attributed to the thicker BGO crystals versus the thinner LSO crystals and the lack of ^176^Lu intrinsic background radiation with LSO crystals, despite the absence of time of flight (1). Other studies have also demonstrated the benefit of LAFOV PET/CT (2,3).

These images demonstrate the potential advantages of the LAFOV digital BGO device in resolving small physiologic and pathologic abnormalities, despite the 79% shorter acquisition time. The smaller recurrence was not visualized on the dynamic images, likely because of the low count and low PSMA uptake, which increased with time. Research continues to define how to best use this new technology, including dynamic or delayed images to improve patient outcomes.

## DISCLOSURE

Michael Hofman acknowledges grant support from the Australian Cancer Research Foundation, National Imaging Facility; a National Collaborative Research Infrastructure Strategy capability, a National Health and Medical Research Council investigator grant, the Prostate Cancer Foundation, and the Peter MacCallum Foundation. GE HealthCare supports installation at the Peter MacCallum Cancer Centre through a device evaluation agreement. Shankar Siva reports support from the Cancer Council Victoria Colebatch Fellowship. No other potential conflict of interest relevant to this article was reported.
